# Solid State NMR: A Powerful Tool for the Characterization of Borophosphate Glasses

**DOI:** 10.3390/molecules25020428

**Published:** 2020-01-20

**Authors:** Grégory Tricot, Lazzat Alpysbay, Bertrand Doumert

**Affiliations:** 1 Université de Lille, CNRS, UMR 8516 - LASIR - Laboratoire de Spectrochimie Infrarouge et Raman, F-59000 Lille, France; lazzat.alpysbay.etu@univ-lille.fr; 2 Université de Lille, CNRS, INRA, Centrale Lille, ENSCL, Univ. Artois, FR 2638 - IMEC - Institut Michel-Eugène Chevreul, F-59000 Lille, France; bertrand.doumert@univ-lille.fr

**Keywords:** MAS-NMR, glasses, borophosphate, structure

## Abstract

This review will show how solid state nuclear magnetic resonance (NMR) has contributed to a better understanding of the borophosphate glass structure. Over the last fifteen years, 1D and 2D magic angle spinning (MAS)-NMR has been used to produce key information about both local and medium range organization in this type of glass. After a brief presentation on borophosphate glasses, the paper will focus on the description of the local order of phosphate and borate species obtained by 1D ^31^P-and ^11^B-MAS-NMR experiments, with a special emphasis on the improvements obtained at high magnetic fields on the borate speciation description. The last part of this review will show how correlation NMR provided new insights into the intermediate length scale order. Special attention will be paid to the quantitative data retrieved from ^11^B/^31^P REDOR-based NMR sequences and to the qualitative connectivity schemes observed on the 2D ^11^B/^31^P maps edited with the heteronuclear multiple quantum coherence (HMQC) NMR techniques.

## 1. Introduction

Glass is ubiquitous in our modern society with numerous applications in everyday life (tableware, windows, etc.) and technical uses (optical fibers, sealing glasses, waste confinement matrixes, coatings, etc.) [1,2,3,4,5,6,7,8]. A large proportion of these glasses are called ‘silicate’ since their structural backbone (called the glass network) is formed by interconnected silica tetrahedra SiO_4_^4-^. However, SiO_2_ is not the only oxide that can produce glasses. V_2_O_5_, As_2_O_5_, TeO_2_, GeO_2_, P_2_O_5_, and B_2_O_3_ also belong to the ‘glass former oxide’ (GFO) category, with the latter two giving rise to the ‘phosphate’ and ‘borate’ glass families, respectively [9,10].

Oxide glasses can be formulated over a wide range of formulations by mixing GFO to other oxides (called modifiers), such as Na_2_O, CaO, or MgO [11,12]. These unconstrained compositions allow for a fine tuning of the macroscopic properties that allows the use of glass in many applications. When a single GFO is used in combination with modifier oxides, the glass backbone is formed by interconnected polyhedra of the same chemical nature, and the glass is categorized as a simple network glass. Interesting results can also be obtained when two (or more) different GFOs are mixed in the same formulation [13,14,15,16,17,18,19,20,21,22,23,24]. In such cases, the glass backbone is formed by interconnected polyhedra of different chemical natures.

One of the best examples of such materials, defined as mixed network glass, is provided by the successful and important technological glass Pyrex^®^ developed in 1915 by the Corning society. High quality glasses with improved thermal and mechanical resistances have been obtained by mixing SiO_2_ and B_2_O_3_ in an 83.0/11.6 molar ratio. In a similar way, unexpected behaviors have been obtained with borophosphate glasses, where the network is formed by interconnected phosphate and borate polyhedra. 

**Figure 1:**
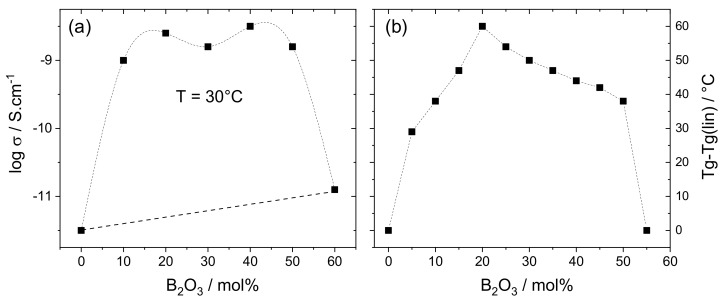
(**a**) Logarithm of conductivity (σ) in the 40Na_2_O-xB_2_O_3_-(60-x)P_2_O_5_ glass system with the values expected from a linear evolution (dotted lines) ([18]); (**b**) Tg-Tg(lin) evolution in the 45Li_2_O-xB_2_O_3_-(55-x)P_2_O_5_ glass system ([19]). Adapted with permission from Elsevier and RSC.

In 1980, Tsuchiya et al. observed a non-linear evolution of the electrical conductivity in the 40Na_2_O-xB_2_O_3_-(60-x)P_2_O_5_ system (x being the molar percent). The glasses were formulated with a constant amount of Na_2_O or Li_2_O (with thus a constant number of charge carriers Na^+^ or Li^+^) and various B_2_O_3_/P_2_O_5_ ratios (denoted as B/P in the following) [18]. Figure 1a clearly shows that the conductivity (σ) values of the mixed network glasses are higher than the values observed in simple network sodium phosphate (x = 0) and sodium borate (x = 60) glasses. It is also noteworthy that all the σ values are higher than the values expected from a linear evolution from the pure phosphate to the pure borate compositions.

This effect, called the mixed glass former effect (MGFE), has been highlighted in different compositions for conductivity [18,20,21] and other macroscopic properties, such as the glass transition temperature (Tg) [7,8,9] or the kinetic fragility [22]. A representative example is given in Figure 1b where the Tg evolution observed in the 45Li_2_O-xB_2_O_3_-(55-x)P_2_O_5_ system is reported [19]. More precisely, the values reported here (denoted as Tg-Tg(lin)) are the difference determined between the measured values (Tg) and the values expected in case of a linear evolution from the pure phosphate to the pure borate composition (Tg(lin)). High Tg-Tg(lin) values thus indicate a strong synergic effect between P and B atoms. This MGFE has contributed to revive the interest for borophosphate glasses and has consequently motivated numerous structural studies [19,20,21,22,23,24,25,26,27,28,29,30,31,32,33,34,35,36,37,38,39,40,41,42,43,44,45]. 

It is now well admitted that the macroscopic properties of these materials are strongly related to their structure and as a consequence, a good understanding of this unexpected and non-linear properties modifications requires a good understanding of the complex structural organization involving the phosphate and borate polyhedra. The first step consists in determining and quantifying the different coordination states of both phosphorus and boron in the glass network. Then, in a second step, special attention has to be paid to the mixing between all these forming blocks. These two steps lead to the determination of local and medium range orders, respectively.

**Figure 2:**
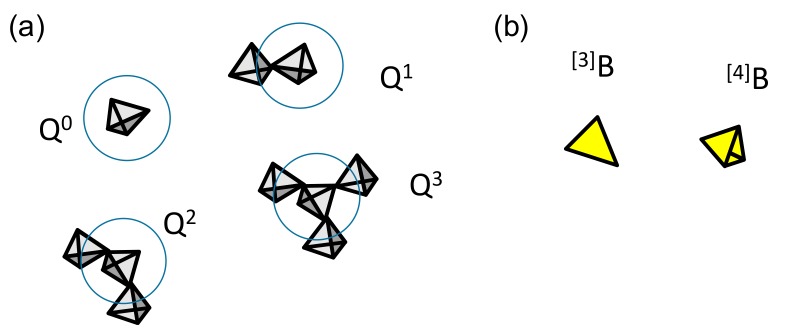
(**a**) Phosphate and (**b**) borate speciation in simple network glasses.

In oxide glasses, the P atoms are always surrounded by four oxygens, forming tetrahedra (Figure 2a). While the nature of these forming blocks is similar for the Si atoms, the +5 charge of P does not allow for the same connectivity scheme and leads to the formation of a P=O linkage that is not available to create P–O–P bonds. As a consequence, P can only create three P–O–P linkages, leading to a maximum polymerization of Q^3^. Boron atoms can adopt two coordination states in oxide glasses and can be surrounded by three or four oxygens, leading to B in a planar triangle (denoted as tri-coordinated or [3]B in the following) and in tetrahedra (denoted as tetra-coordinated or [4]B in the following) (Figure 2b). A critical structural parameter is thus the proportion of tri- and tetra-coordinated borates present in the glass network. This parameter is expressed through the N^4^ values which is the ratio between [4]B and the total B (N4=[4]B/[4]B+[3]B)).

In the following section, we will show how 1D magic angle spinning (MAS) nuclear magnetic resonance (NMR) provides information about the nature and proportion of the forming polyhedra (leading to the description of the local order) and how the correlation NMR provides data onto the connectivity between all these building blocks (leading to the intermediate length scale order determination).

## 2. Description of the Local Order by 1D ^31^P-and ^11^B-MAS-NMR

### 2.1. Nuclear Properties

The nuclear properties (spin, natural abundance, Larmor frequency, and relative sensitivity) of the three NMR sensitive isotopes of boron and phosphorus are reported in Table 1.

Phosphorus atoms can be easily analyzed by NMR, owing to the ^31^P high sensitivity deriving from its 100% natural abundance and high Larmor frequency. ^31^P is a spin-1/2 and is not subjected to the quadrupolar interaction. The ^31^P-NMR signals are thus expected to be symmetric and the spectra decomposition can be carried out using Gaussian peaks in our glasses where the signal lineshape is dominated by the chemical shift distribution. The only limitation of ^31^P for the NMR analysis is its long longitudinal relaxation time (up to minutes) that could lead to very long experimental times, especially when a π/2 flip angle is used for the excitation. Boron atoms can be analyzed through two isotopes that are ^10^B and ^11^B. Both are quadrupolar and are thus subjected to the quadrupolar interaction. Complex shape and asymmetric peaks are thus expected for the boron resonances.

The better sensitivity and lower quadrupolar moment make, of ^11^B, a much more efficient isotope for NMR characterization. The relaxation time of ^11^B is rather short (from a few seconds to a few tens of second) and does not lead to long experimental time especially when short pulses are applied. Special attention has to be paid to the ^11^B signal coming from the probehead, which usually contains a B-containing stator (BN, Macor^®^, etc). Special equipment with B free materials (MgO, Vespel^®^, etc) or systematic background signal substration are necessary to provide quantitative NMR spectra that will only reflect a ^11^B signal coming from the investigated samples. While most of the papers focus on ^11^B to analyze the borate speciation, a few papers used ^10^B to probe the structure of borate glasses [46,47]. To our knowledge, no paper has been published on ^10^B-NMR applied to borophosphate glass.

### 2.2. B MAS-NMR

**Figure 3:**
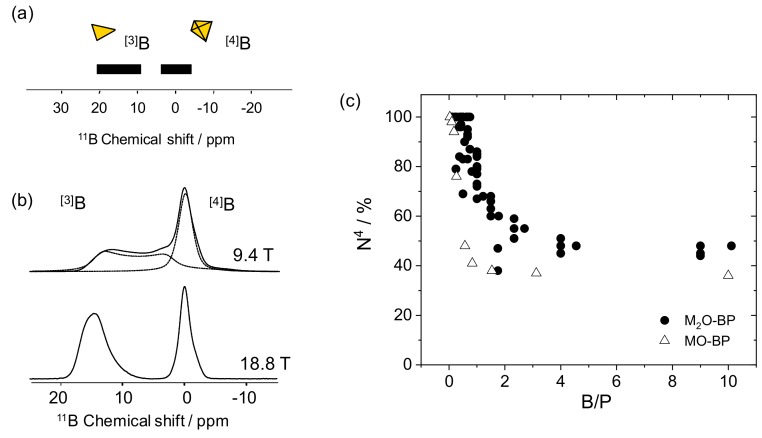
(**a**) Chemical shift ranges corresponding to the [3]B and [4]B speciation. (**b**) ^11^B-MAS-NMR analysis performed on a sodium boro-phosphate glass at 9.4 and 18.8 T. (**c**) Compilation of N^4^ values observed in different mono- (full circles) and di- (empty triangles) valent ions containing borophosphate glasses [19,20,21,22,23,24,28,29,31,32,34,35,36] versus the glass composition expressed here with the B_2_O_3_/P_2_O_5_ ratio (B/P) ratio.

As previously mentioned, boron is present in glass networks as [3]B and [4]B species. This difference in the number of surrounding oxygens induces a strong difference in the chemical environment and consequently in the chemical shift values. Each species is characterized by a given chemical shift range, with the lower coordination state ([3]B) leading to the more deshielded (more positive) chemical shift values. As reported in Figure 3a, tri-coordinated boron chemical shift can be found between 20 and 10 ppm whereas tetra-coordinated boron chemical shift values are present between 5 and −5 ppm.

The quadrupolar interaction intensity (and the NMR signal broadness) can also be used to differentiate the [3]B and [4]B signals. This interaction depends on two parameters, which are the quadrupole moment (Table 1) and the electric field gradient (efg). While the first parameter is characteristic of the nucleus and is thus similar for both [3]B and [4]B species, the efg parameter is the signature of chemical environment asymmetry and presents higher values in the case of more asymmetric [3]B species. As a consequence, [3]B species experience a larger quadrupolar interaction leading to asymmetric and broad signals.

The quadrupolar constant values (C_Q_) determined for [3]B signals reach 2.5–2.9 MHz [22,23,24,30] whereas the [4]B signal only experiences a weak C_Q_ of 0.4–0.6 MHz [29]. It is noteworthy that this signal, whose lineshape is usually dominated by the chemical shift distribution, can be treated with a standard Gaussian/Lorentzian model.

The final consequence of the quadrupolar interaction is illustrated in Figure 3b with two ^11^B-MAS-NMR experiments performed on the same sample at two different static fields. At standard field (9.4 T), the two signals overlap due to the important broadness of the [3]B signals due to the quadrupolar interaction. Quantification between the two borate species thus requires a decomposition of the experimental data with proper models [48]. However, this decomposition introduces a small error in the quantification and easier, faster, and more accurate quantification can be achieved with ^11^B-MAS-NMR measurements performed at high field, as demonstrated by the spectrum obtained at 18.8 T (Figure 3b).

The high static field allows for a strong reduction of the effect of the quadrupolar interaction. Consequently, the broadness of the [3]B signal decreases at high field and experiments performed at B0 > 14.1 T present clearly separated [3]B and [4]B regions. Quantification can then be obtained by signal integration on ^11^B-NMR spectra. It is noteworthy that a rigorous quantification requires the NMR spectra to be first corrected from the satellite transition contribution. The superiority of high field NMR has been used in many studies to obtain efficient borate quantification [22,23,24,33]. However, it is noteworthy that advanced NMR sequences like multiple quantum [49] or satellite [50] transition (MQ- and ST-) MAS techniques can be used to improve the resolution owing to the editing of a 2D spectrum. While improved resolution can be obtained in the indirect dimension, no direct quantification is possible from these 2D spectra.

Figure 3c gathers different borate species quantifications obtained by ^11^B-NMR on several M_2_O-B_2_O_3_-P_2_O_5_ and MO-B_2_O_3_-P_2_O_5_ systems [19,20,21,22,23,24,28,29,31,32,34,35,36]. The N^4^ parameter has been used to characterize the borate speciation modifications with the composition, this latter being expressed here with the molar B_2_O_3_/P_2_O_5_ ratio (B/P). A general behavior can be observed with a constant decrease of the N^4^ parameter when the B/P ratio increases. Borons thus enter in the phosphate network as [4]B units (low B/P ratio) and are replaced by trigonal borons when B_2_O_3_ amounts increase (high B/P ratio).

The complete set of data suggests that the borate speciation starts to be dominated by planar groups for B/P values of 3. It is also striking to note that even at a very high B/P ratio, the N^4^ values seem to reach a plateau around 40%. Boro-phosphate glasses show a different behavior than borate or borosilicate glasses, for which the borate speciation is mainly governed by the presence of M_2_O or MO oxides that play the role of charge compensators. Here, the +5 charge of the P atoms is sufficient to compensate the +3 charge of B atoms in mixed borophosphate structural units and allows for tetrahedral borons to be formed as in the crystalline BPO_4_ structure. It is also noteworthy that the presence of borons in 4-fold coordination in the low B/P ratio glasses is in line with results obtained on aluminophosphate glasses in which Al atoms also enter in the network under its higher coordination state ([6]Al) before being replaced by [5]Al and [4]Al units [51]. 

Beyond providing an easy and efficient quantification of the tetra- and tri-gonal borate species, the high field NMR experiments also afford a better description of these borate species, particularly in the case of the [4]B units. The recent experiments performed at very high field produced spectra presenting an improved resolution in the [4]B region and allowing, thus, for a better description of the tetra-coordinated borate species [22,23,24]. Before the availability of high field NMR spectrometer, the low resolution in the [4]B region did not permit an efficient analysis of the NMR data and the borate speciation was often reduced to the presence of B(OP)_4_ groups, in good agreement with the results obtained by Ray et al. [52] in 1975 from Raman measurements. The very low number of available crystalline borophosphate compounds also prevents efficient comparison of the chemical shift values obtained on the vitreous systems.

**Figure 4:**
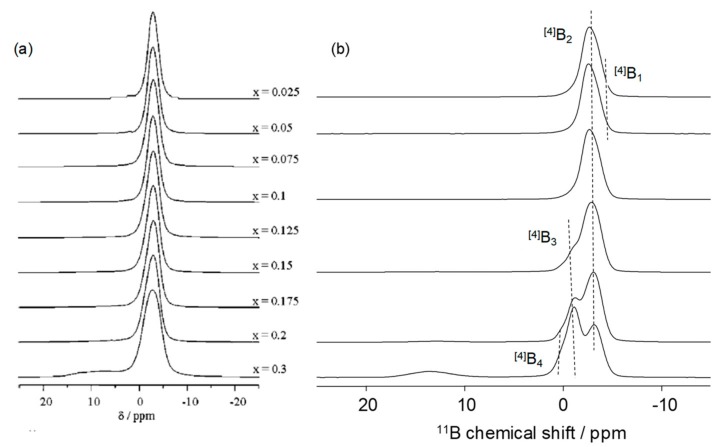
(**a**) ^11^B-MAS-NMR spectra obtained on xB_2_O_3_-(1-x)NaPO_3_ glasses at 11.7 T [14] and (**b**) 18.8 T. Part (**a**), reprinted with permission from ACS.

Figure 4 presents the ^11^B MAS-NMR spectra obtained on the xB_2_O_3_-(100-x)NaPO_3_ system at 11.7 [14] and 18.8 T. In the study performed at 11.7 T, the signal observed around -3 ppm for the samples containing up to 30 mol% of B_2_O_3_ is attributed to the presence of a B(OP)_m_ species with m = 3 or 4. At B_2_O_3_ amounts higher than 30%, the authors observed the appearance of trigonal borate and explain the increase of the [4]B signal broadness by the presence of several [4]B peaks with close chemical shift values. The same system has been recently re-investigated at higher field (18.8 T) (Figure 4b).

While the data of the previous articles are fully supported by the recent high-field NMR analysis, the better resolution allows for refining of some of the above-mentioned conclusions. At least four different tetra-coordinated borate species can be distinguished all along the composition line (denoted as [4]B_1–4_ in Figure 4b) confirming the complex nature of the tetra-coordinated speciation suggested in the previous study. This behavior is general and high field NMR analysis usually reveals a very complex borate speciation. Unfortunately, the exact nature of all these signals is not yet perfectly clear and 1D ^11^B MAS-NMR needs to be completed by correlation techniques, as will be shown in the next section of the article.

### 2.3. P-MAS-NMR

**Figure 5:**
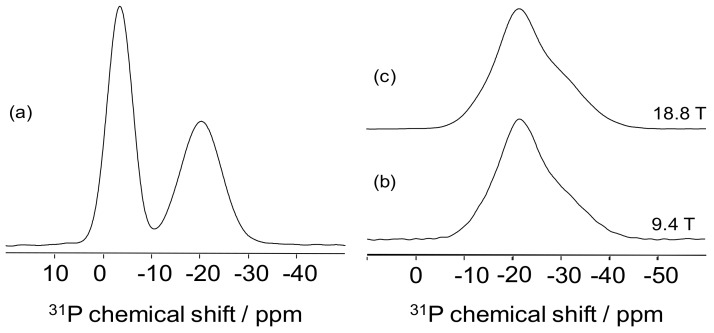
^31^P-MAS-NMR spectra obtained (**a**) on a lithium phosphate at 9.4 T and (**b**–**c**) on lithium boro-phosphate glasses at (**b**) 9.4 T and (**c**) 18.8 T.

^31^P MAS-NMR is a very efficient tool for the description of simple network phosphate glass structure. Different chemical shift ranges are observed depending on the number of connected phosphorus. The 1D ^31^P MAS-NMR can then be used to distinguish and quantify the different Q^n^ species present in the simple network glass (Figure 2a) [53]. It is noteworthy that only four species (Q^3^, Q^2^, Q^1^, and Q^0^) can co-exist in this type of glass. A very simple example is given in Figure 5a with the ^31^P NMR spectrum obtained on a 45Li_2_O-55P_2_O_5_ glass. Two different signals are observed including a peak at –20 ppm, corresponding to Q^2^ species and a resonance at –2 ppm, corresponding to Q^1^ species [53]. Quantification is then achieved by determining the relative proportions between the two resonances.

The ^31^P MAS-NMR spectrum of lithium borophosphate appears much more complex to analyze. No clear distinction and numbering can be done from the 1D experiments. In the case of this mixed network glass, the number of possible phosphate species reaches 34 instead of the four expected in the simple network phosphate glass. This high number is due to all the possible configurations involving linkages between P and trigonal or tetragonal borate units. The standard Q^n^ notation where n is the number of connected P (or the number of bridging oygen in case of the simple network glass) is thus not capable of describing such species.

An improved notation Q^n^_m[x]B_ has to be used to be able to differentiate all the phosphate species using the number of connected P (n) and borate (m), the total number of bridging oxygens being obtained by adding n and m. 

While it is unlikely than the 34 species co-exist in the same glass structure, the boro-phosphate network is however composed by different species with close chemical shift values. The consequence of this complex speciation is the very broad signal observed in Figure 5b that results from the superimposition of these different signals with close chemical shift values. ^11^B MAS-NMR experiments have shown that increasing the static field leads to a resolution improvement. As illustrated in Figure 5b,c, increasing B_0_ does not lead to any resolution improvement for the ^31^P MAS-NMR experiment. In this case, the broadness is due to the chemical shift distribution that increases with the static field. At this point, ^31^P MAS-NMR spectra appear thus limited for the structural characterization of the borophosphate network and are usually interpreted only through the overall chemical shift modification [54]. A deeper analysis, however, permits additional information provided by correlation NMR, as will be discussed in the next section.

## 3. New Insights Provided by ^11^B/^31^P Correlation NMR

Different correlation NMR sequences are available to investigate the structure of glasses. The choice of the proper sequence to apply is related to the nature of the chemical information, which is expected. When interaction between two polyhedra of the same chemical nature (in our case ^11^B/^11^B or ^31^P/^31^P) is analyzed, homo-nuclear correlation NMR sequences have to be used. When the mixing between two polyhedra of different chemical natures is investigated (^11^B/^31^P), hetero-nuclear sequences have to be applied. These interactions can then be analyzed through the chemical connectivity or the spatial proximity point of views by using scalar- or dipolar- based correlation NMR sequences.

In most cases, dipolar sequences present a better sensitivity than the scalar techniques, but two nuclei that are close spatially are not necessarily connected [33]. As a consequence, dipolar correlation should be treated with caution. Scalar based NMR sequences are usually less sensitive and are also based on spin-echo sequences, which restrict their use to samples with long transverse relaxation time. A lack of signal for short transverse relaxation time samples is thus not a definitive and unambiguous evidence of the absence of interaction.

**Figure 6:**
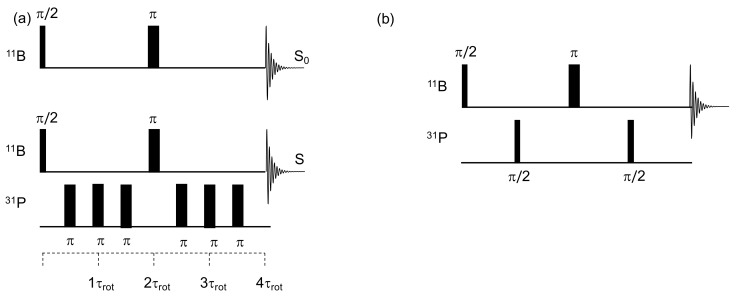
(**a**) ^11^B(^31^P) rotational echo double resonance (REDOR) and (**b**) ^11^B(^31^P) heteronuclear multiple quantum coherence (HMQC) pulse sequences.

Borophosphate glasses have been widely investigated by correlation NMR. In the following section are gathered the most important studies based on rotational echo double resonance (REDOR) [55,56] and heteronuclear multiple quantum coherence (HMQC) [57] correlation NMR sequences (Figure 6) that provide quantitative and qualitative description of the ^11^B/^31^P interactions found in the glass network.

### 3.1. REDOR Based Sequences

Borophosphate glasses have been extensively studied with REDOR based NMR techniques [25,26,29,30,31]. The dipolar mediated sequence, sketched in Figure 6a, is used to quantify the ^11^B/^31^P heteronuclear interaction. The basic principle of the ^11^B(^31^P) REDOR experiment is to record two sets of signals. The first set (called S_0_) is recorded using a ^11^B rotor-synchronized spin echo, during which the MAS conditions suppress the heteronuclear dipolar interaction. The second set of data (called S) is recorded under similar conditions; however, the dipolar interaction is reintroduced through the application of π-pulses on the ^31^P channel every half and full rotor period (τ_rot_) (Figure 6a shows the sequence used with a total of four full rotor periods).

The evolution of the normalized difference ((S_0_-S)/S_0_ = ΔS/S_0_) versus the complete echo delay (and thus the number of applied π-pulses) can be used to quantify the dipolar interaction. Analytic expression is available in the case of isolated B-P pairs, however, is not suitable for glasses that involve multi-spin systems [56]. In this case, the dipolar interaction depends on the B–P distance but also on the number of phosphorus surrounding each borate species. In the studies using REDOR on boro-phosphate systems, the multi-spin approximation has been used and only the beginning of the curve is fitted (ΔS/S_0_ < 0.2) with the parabolic function reported below:

(1) $$\frac{∆S}{S0}=\frac{4}{3\pi^{2}}\times {\left(NTr\right)}^{2}\times M_{2}$$

with NTr being the complete echo delay and M_2_ the Van Vleck second moment. This latter can be related to the number of phosphorous attached to each boron species, if a constant distance is assumed [56]. In case of borophosphate systems, it has been found that one B–O–P linkage leads to a M_2_ values of 4.5 10^6^ s^−2^ [25,29,30,31].

**Figure 7:**
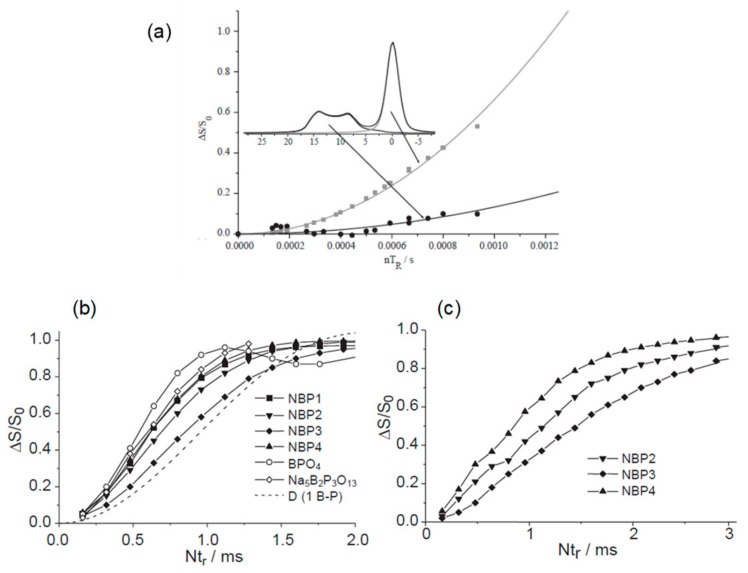
(**a**) ^11^B(^31^P) REDOR curves obtained on [4]B and [3]B species on a Ag_2_O-B_2_O_3_-P_2_O_5_ glass [25]; (b-c) ^11^B(^31^P) REDOR curves observed for (**b**) [4]B and (c) [3]B on different sodium borophosphate glasses [26]. Reprinted with permission from Elsevier.

The two first applications of ^11^B(^31^P) REDOR NMR to borophosphate glasses were performed in 2005 [25,26] and are reported in Figure 7. Applied on silver borophosphate (Figure 7a), REDOR measurements show that the tetra-coordinated borate species differ by the number of attached P. The different [4]B units distinguished on the 1D MAS-NMR experiment, performed at 11.7 T, were annotated using the B(OP)_p_ nomenclature and the deshielded signals originated from a lower number of attached phosphorus [25]. The results also ruled out the presence of B(OP)_4_ groups in the glass network, whereas previous studies usually concluded for the presence of this structural unit in the glass network.

Finally, the ^11^B(^31^P) REDOR results did not show any particular interaction between the phosphate units and the tri-coordinated borons, from which the absence of the P–O–[3]B linkage was finally suggested. This last conclusion was mediated by the second REDOR study performed on sodium boro-phosphate glasses (Figure 7b,c) [26]. While the REDOR results reported in Figure 7b indicate that [4]B groups are attached to three phosphorus atoms and that this number decreases with the B_2_O_3_ content of the glasses, significant interaction between the trigonal borate and phosphate was observed in this system (Figure 7c).

The authors finally concluded that some linkages between the trigonal and the phosphate moities may exist in the glass matrix. However, this type of linkage was not highlighted by the 2D map edited with the CP-HETCOR sequence, as it will be discussed in the next subsection. More recently, ^11^B(^31^P) REDOR was used to analyze the structure of alkali borophosphate glasses and confirmed the previous assignments including the presence of B(OP)_3,4_ units at low B_2_O_3_ amounts that is replaced then by borate connected to a lower number of P atoms [19]. It is noteworthy that some of the previous studies also try to determine the P(OB)_q_ speciation by using the ^31^P(^11^B) REDOR NMR sequence modified to take into account the less efficient excitation of the quadrupolar ^11^B nuclei by π-pulse scheme [19,25,29,30].

### 3.2. D ^11^B/^31^P Correlation Maps

In addition to the quantitative data retrieved from the REDOR based sequences, the borophosphate organization has also been investigated by qualitative correlation maps. The first editing of a 2D map giving direct access to the B/P connectivity scheme was performed in 2005 [26] using the standard cross polarisation sequence. In this widely used technique, the magnetization is first created through a π/2 pulse applied to ^11^B and then transferred from ^11^B to ^31^P, by irradiating both channels simultaneously. An efficient transfer is achieved when the two irradiations fulfill the Hartmann–Hahn conditions [58,59]. This transfer is mediated through the dipolar interaction and is thus dependent to the distance (d) between the two atoms (~1/d^3^).

**Figure 8:**
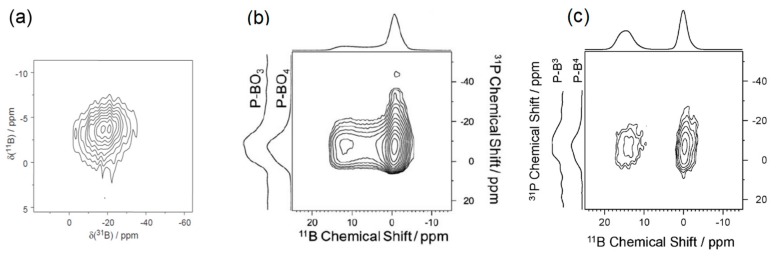
^11^B/^31^P 2D maps obtained on (**a**) sodium borophosphate glass at 9.4 T with the CP-HETCOR sequence, (**b**) lithium borophosphate glass at 9.4 T with the D-HMQC and (**c**) lithium borophosphate glass at 18.8 T with the J-HMQC NMR techniques [19,26,33]. Reprinted with permission from Elsevier and RSC.

Figure 8a shows the results obtained in 2005 for a sodium borophosphate glass. The 2D map is displayed with ^31^P and ^11^B dimensions in the horizontal and vertical axes, respectively. The correlation signal indicates interaction between the phosphate units and tetra-coordinated boron. The spatial proximity highlighted here has then been discussed in terms of chemical connectivity. This 2D map is the first experimental evidence of the presence of mixed [4]B–O–P linkages in the glass network. While the lack of correlation signal involving the trigonal borons and phosphate sites could be related to the absence of [3]B–O–P linkages, the authors indicated that the cross polarisation technique was not suitable to highlight this kind of correlation. Indeed, the magnetization transfer is much more complex to optimize, due to the strong quadrupolar interaction experienced by the trigonal borate units.

The debate about the presence or absence of [3]B–O–P bonds in borophosphate was definitively closed in 2015, owing to HMQC NMR experiments (Figure 6b) [33]. This technique is based on a spin-echo performed on the ^11^B channel, which is modulated by two π/2 pulses applied to the ^31^P channel. These two π/2 pulses create scalar heteronuclear coherences, especially when the echo delay is correctly set up (τ = 1/2J_P–B_). This scalar version was modified in 2005 to become a dipolar mediated NMR sequence. P/B dipolar interaction is then reintroduced through two pulse schemes applied to the ^31^P channel. [60,61,62].

In 2010, the dipolar HMQC was applied on a 9.4 T NMR spectrometer to lithium borophosphate glasses (Figure 8b) to edit a 2D map with ^11^B and ^31^P dimensions as horizontal and vertical axes [19]. The 2D map is also accompanied by ^11^B and ^31^P 2D map projections in the horizontal and vertical axes, respectively. In addition to the main correlation signal involving [4]B and phosphate species, which confirms all the previous assignments, an unexpected signal was observed between [3]B and phosphate units. This signal showing close proximity between trigonal borate and phosphate species suggested the presence of [3]B–O–P linkages in the glass network. This result was confirmed in 2015 by the acquisition of scalar mediated HMQC experiment (Figure 8c) [33]. Beyond clearly showing the presence of [3]B–O–P bonds in the glass network, the high field used for the experiment produced a well resolved 11B dimension 2D map with two correlation signals corresponding to [3]B–O–P and [4]B–O–P linkages.

Editing of 2D ^11^B/^31^P maps was also used in different studies to help in the 1D ^31^P MAS-NMR spectra decomposition [19,22,23,33]. As mentioned in Section 2.3, the ^31^P MAS-NMR spectra obtained on borophosphate glasses are very broad, and an efficient and trustworthy decomposition cannot be performed on the basis of the 1D NMR data only. The editing of 2D ^11^B/^31^P maps allows for distinguishing the different types of phosphorus connected to boron in the indirect dimension of the 2D maps. Then, a comparison between the extracted ^31^P spectra (showing all the phosphorus connected to boron) and the 1D ^31^P MAS-NMR spectra (showing phosphorus connected to boron and non-connected to boron) helps achieve a better decomposition of the 1D NMR data.

**Figure 9:**
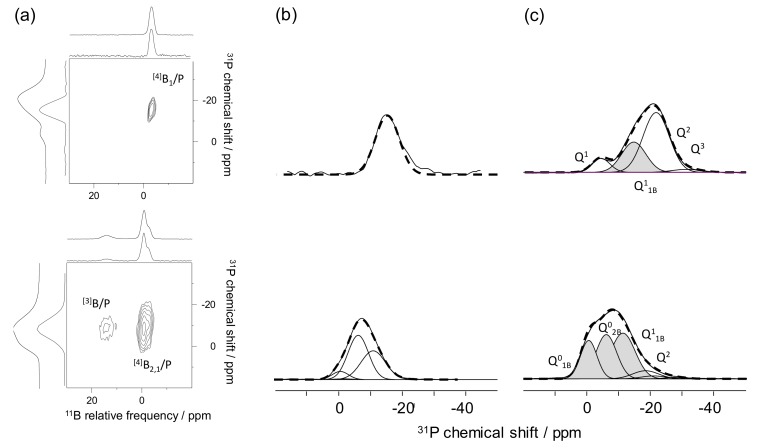
(**a**) ^11^B/^31^P 2D maps obtained on lithium borophosphate glasses with low (top) and high (bottom) B_2_O_3_ content at 18.8 T with the D-HMQC NMR sequence, (**b**) ^31^P projections of the 2D map showing the phosphate attached to borate and (**c**) 1D ^31^P–MAS-NMR spectra accompanied by the decompositions supported by the 2D NMR data [22]. Adapted with permission from RSC.

A representative example is given in Figure 9a with the 2D maps obtained on a 50Li_2_O-5B_2_O_3_-45P_2_O_5_ (top) and 50Li_2_O-25B_2_O_3_-25P_2_O_5_ (bottom) glass [22]. The 2D maps are accompanied by the ^31^P projections and the 1D ^31^P MAS-NMR experiments on the vertical axes. The ^31^P projections of the 2D maps are reported in Figure 9b and are the signatures of the phosphate moieties involved in P/B interaction. These projections have been decomposed using a minimum number of components, with each component characterized by a chemical shift and a broadness value. Finally, all these values were used as input parameters to produce a supported decomposition of the 1D ^31^P MAS-NMR spectra (Figure 9c). In the decompositions the signals corresponding to phosphate attached to B (in grey) and the signals corresponding to phosphate not attached to borate units (in white) are separated.

This methodology overcame the poor resolution of the ^31^P MAS-NMR experiments and provided information about the number of phosphate species and their chemical nature [22]. It is noteworthy that the determination of the different P(OB)_q_ units derived from both 2D maps and REDOR based sequences will be discussed in a forthcoming paper and compared to the data obtained on the other important mixed network phosphate glasses, including the aluminophosphate system.

## 4. Conclusions

In this review, we showed how 1D/2D solid state NMR contributes to a better understanding of the borophosphate network structure. 1D ^11^B MAS-NMR appears to be the technique of choice to highlight the presence of borate species under its tri- ([3]B) or tetra- ([4]B) coordinated configurations, especially at high field, when the [3]B and [4]B signals are clearly separated. Being quantitative, the 11B NMR experiments can also provide accurate determination of the relative proportions between the two species. While a deep analysis of the [3]B region is still missing, the presence of different [4]B sites has been highlighted, owing to the optimized resolution of that region offered by the high field NMR analysis. In spite of the excellent ^31^P NMR characteristics, 1D ^31^P MAS-NMR analysis of borophosphate glasses offers only poorly resolved spectra, due to the presence of many overlapping components, from which clear numbering and identification is not possible. 

Correlation NMR has proven to be an efficient technique providing information about the intermediate length scale order. In this review, we focused on REDOR based NMR sequences, which provide quantitative information about the number of P attached to the borate units.

To complete the structural model, we showed that qualitative 2D ^11^B/^31^P maps showing the presence of P–O–B linkages could be edited with the J/D- HMQC NMR techniques. In addition, the distinction between P attached to B and P not attached to B can be achieved with the 2D maps, and this aids in the decomposition of the broad and uninformative ^31^P MAS-NMR spectra. Altogether the data allows for a better description of the borophosphate network in many systems.

Improvement will be certainly obtained in the next years owing to the ^11^B DQ-SQ NMR technique [63] that allows monitoring for the presence and nature of the ^[x]^B–O–^[x]^B bonds. This technique, based on the dipolar interaction, provides 2D maps with signals showing spatial proximity. The relevance of discussing these signals in terms of chemical connectivity is still under question. We believe that the significant progress recently made in borosilicate glasses, with the help of molecular dynamics [64], will be quickly transfered to the borophosphate glasses and will provide a definitive answer. ^31^P/^31^P correlation NMR could also be used to better describe the interactions between the different phosphate species. 

Structural model improvements would also be possible through oxygen NMR experiments. Being the most abundant nucleus in oxide glasses, oxygen is thus a potential source of crucial information and could be used, in our case, to differentiate and quantify P–O–P, P–O–^[x]^B, and even ^[x]^B–O–^[x]^B linkages. Unfortunately, the NMR sensitive isotope ^17^O does not present favorable NMR characteristics: its low natural abundance (0.037%) thus requires ^17^O enrichment, and its 5/2 spin requires special NMR sequences to provide spectra with sufficient resolution. While ^17^O has been widely used to analyze the structure of borosilicate-based glasses [65,66,67,68,69,70], there is, to our knowledge, only one paper about ^17^O NMR on enriched borophosphate glasses [26]. However, this paper from 2005 clearly showed the possibility of separating bridging P–O–P and P–O–B oxygens by using MQ-MAS NMR experiments [49] and there is no doubt that conducting such a study with the high field NMR machines and the correlation NMR sequences now available would provide a very interesting set of data that would shed new light onto the borophosphate network organization.

**Figure 10:**
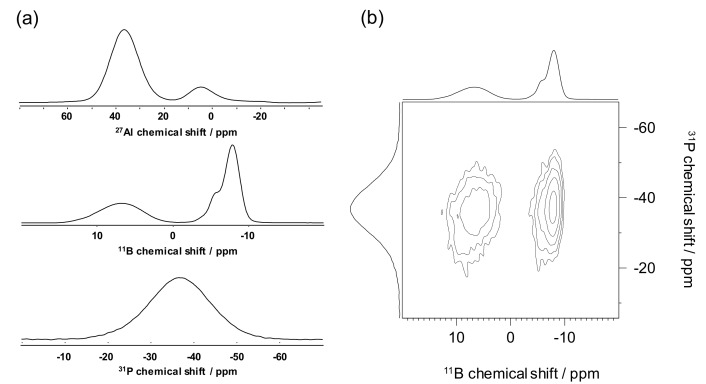
(**a**) 1D ^27^Al, ^11^B and ^31^P, (**b**) 2D ^11^B(^31^P) D-HMQC obtained at 18.8 T obtained on Al_2_O_3_-B_2_O_3_-P_2_O_5_ glass.

Finally, we believe that this overall methodology will then be applied to much more complex systems, especially when a borophosphate network is accompanied by another GFO like SiO_2_ [71] or Al in four-fold coordination. As shown in Figure 10, the extent of mixing in a modifier free Al_2_O_3_-B_2_O_3_-P_2_O_5_ glass can be efficiently analyzed with ^11^B, ^27^Al and ^31^P 1D NMR spectra and the ^11^B(^31^P) 2D map.

## Figures and Tables

**Table 1:** Nuclear properties of ^31^P, ^10^B, and ^11^B isotopes. Quadrupolar moment (eQ), natural abundance (Nat. abund.), and Larmor frequency (Larmor freq.).

Isotopes	Spin	eQ/10^−3^ barns	Nat. Abund./%	Larmor freq./MHz.T^−1^	Sens. (^13^C = 1)
31P	1/2	-	100.0	17.22	379
10B	3	84.7	19.9	5.57	1.4
11B	3/2	40.7	80.1	13.65	151.3

## References

[1] Maurer R. Glass fibers for optical communications. Proceedings of the IEEE.

[2] Ballato J., Dragic P. (2016). Glass: The Carrier of Light - A Brief History of Optical Fiber. Int. J. Appl. Glas. Sci..

[3] Dragic P.D., Cavillon M., Ballato J. (2018). Materials for optical fiber lasers: A review. Appl. Phys. Rev..

[4] Donald I.W., Mallinson P.M., Metcalfe B.L., Gerrard L.A., Fernie J.A. (2011). Recent developments in the preparation, characterization and applications of glass- and glass–ceramic-to-metal seals and coatings. J. Mater. Sci..

[5] Brow R.K., Tallant D.R. (1997). Structural design of sealing glasses. J. Non-Cryst. Solids.

[6] Lee W.E., Ojovan M.I., Stennett M.C., Hyatt N.C. (2006). Immobilisation of radioactive waste in glasses, glass composite materials and ceramics. Adv. Appl. Ceram..

[7] Sengupta P. (2012). A review on immobilization of phosphate containing high level nuclear wastes within glass matrix – Present status and future challenges. J. Hazard. Mater..

[8] Gin S., Jollivet P., Tribet M., Peuget S., Schuller S. (2017). Radionuclides containment in nuclear glasses: an overview. Radiochim. Acta.

[9] Zachariasen W.H. (1932). The Atomic Arrangement in Glass. J. Am. Chem. Soc..

[10] Sun K.-H. (1947). Fundamental condition of glass formation. J. Am. Ceram. Soc..

[11] Zarzycki J. (1991). Glasses and the vitreous state.

[12] Shelby J.E. (2005). Introduction to glass science and technology.

[13] Turner W.E.S. (1924). The use of boric oxide in glass-making. J. Am. Ceram. Soc..

[14] Nordberg M.E. (1944). Properties of some vycor-brand glasses. J. Am. Ceram. Soc..

[15] Abe T. (1952). Borosilicate glasses. J. Amer. Ceram. Soc..

[16] Haller W., Blackburn D.H., Wagstaff F.E., Charles R.J. (1970). Metastable immiscibility surface in the system Na2O-B2O3-SiO2. J. Amer. Ceram. Soc..

[17] Peng S., Ke Z., Cao X., Shan C., Zhao F., Guan M., Shi L., Sun Y., Yang Y., Ma L. (2020). A novel type of borosilicate glass with excellent chemical stability and high ultraviolet transmission. J. Non-Crystalline Solids.

[18] Tsuchiya T., Moriya T. (1980). Anomalous behavior of physical and electrical properties in borophosphate glasses containing R2O and V2O5. J. Non-Crystalline Solids.

[19] Raguenet B., Tricot G., Silly G., Ribes M., Pradel A. (2011). Revisiting the ‘mixed glass former effect’ in ultra-fast quenched borophosphate glasses by advanced 1D/2D solid state NMR. J. Mater. Chem..

[20] Magistris A., Chiodelli G., Villa M. (1985). Lithium borophosphate vitreous electrolytes. J. Power Sources.

[21] Zielniok D., Cramer C., Eckert H. (2007). Structure/Property Correlations in Ion-Conducting Mixed-Network Former Glasses: Solid-State NMR Studies of the System Na2O−B2O3−P2O5. Chem. Mater..

[22] Muñoz-Senovilla L., Tricot G., Muñoz F. (2017). Kinetic fragility and structure of lithium borophosphate glasses analysed by 1D/2D NMR. Phys. Chem. Chem. Phys..

[23] Tricot G., Saitoh A., Takebe H. (2015). Intermediate length scale organisation in tin borophosphate glasses: new insights from high field correlation NMR. Phys. Chem. Chem. Phys..

[24] Raguenet B., Tricot G., Silly G., Ribes M., Pradel A. (2012). The mixed glass former effect in twin-roller quenched lithium borophosphate glasses. Solid State Ionics.

[25] Elbers S., Strojek W., Koudelka L., Eckert H. (2005). Site connectivities in silver borophosphate glasses: new results from 11B{31P} and 31P{11B} rotational echo double resonance NMR spectroscopy. Solid State Nucl. Magn. Reson..

[26] Zeyer-Düsterer M., Montagne L., Palavit G., Jäger C. (2005). Combined 17O NMR and 11B–31P double resonance NMR studies of sodium borophosphate glasses. Solid State Nucl. Magn. Reson..

[27] Storek M., Böhmer R., Martin S.W., Larink D., Eckert H. (2012). NMR and conductivity studies of the mixed glass former effect in lithium borophosphate glasses. J. Chem. Phys..

[28] Qiu D., Guerry P., Ahmed I., Pickup D.M., Carta D., Knowles J.C., Smith M.E., Newport R.J. (2008). A high-energy X-ray diffraction, 31P and 11B solid-state NMR study of the structure of aged sodium borophosphate glasses. Mater. Chem. Phys..

[29] Raskar D., Rinke M.T., Eckert H. (2008). The Mixed-Network Former Effect in Phosphate Glasses: NMR and XPS Studies of the Connectivity Distribution in the Glass System (NaPO3)1−x(B2O3)x. J. Phys. Chem. C.

[30] Rinke M.T., Eckert H. (2011). The mixed network former effect in glasses: solid state NMR and XPS structural studies of the glass system (Na2O)x(BPO4)1-x. Phys. Chem. Chem. Phys..

[31] Larink D., Eckert H., Reichert M., Martin S.W. (2012). Mixed Network Former Effect in Ion-Conducting Alkali Borophosphate Glasses: Structure/Property Correlations in the System [M2O]1/3[(B2O3)x(P2O5)1–x]2/3 (M = Li, K, Cs). J. Phys. Chem. C.

[32] Christensen R., Olson G., Martin S.W. (2013). Structural Studies of Mixed Glass Former 0.35Na2O + 0.65[XB2O3 + (1 – x)P2O5] Glasses by Raman and 11B and 31P Magic Angle Spinning Nuclear Magnetic Resonance Spectroscopies. J. Phys. Chem. B.

[33] Tricot G., Raguenet B., Silly G., Pradel A., Ribes M., Eckert H. (2015). P–O–B 3 linkages in borophosphate glasses evidenced by high field 11 B/ 31 P correlation NMR. Chem. Commun..

[34] Sdiri N., Elhouichet H., Elakermi E., Dhifallah A., Ferid M. (2015). Structural investigation of amorphous Na2O–P2O5–B2O3 correlated with its ionic conductivity. J. Non-Crystalline Solids.

[35] Michaelis V.K., Kachhadia P., Kroeker S. (2013). Clustering in borate-rich alkali borophosphate glasses: A 11B and 31P MAS NMR study. Phys. Chem. Glass..

[36] Ducel J., Videau J. (1992). Physical and chemical characterizations of sodium borophosphate glasses. Mater. Lett..

[37] Lim J., Schmitt M., Brow R., Yung S. (2010). Properties and structures of tin borophosphate glasses. J. Non-Crystalline Solids.

[38] Tricot G., Ben Tayeb K., Koudelka L., Mošner P., Vezin H. (2016). Insertion of MoO3 in Borophosphate Glasses Investigated by Magnetic Resonance Spectroscopies. J. Phys. Chem. C.

[39] Zhang L., Eckert H. (2005). Synthesis and structural evolution of Al2O3–B2O3–P2O5gels and glasses. J. Mater. Chem..

[40] Yu Y., Stevensson B., Edén M. (2017). Medium-Range Structural Organization of Phosphorus-Bearing Borosilicate Glasses Revealed by Advanced Solid-State NMR Experiments and MD Simulations: Consequences of B/Si Substitutions. J. Phys. Chem. B.

[41] Carta D., Qiu D., Guerry P., Ahmed I., Neel E.A.A., Knowles J.C., Smith M.E., Newport R.J. (2008). The effect of composition on the structure of sodium borophosphate glasses. J. Non-Crystalline Solids.

[42] Mascaraque N., Durán A., Munoz F. (2011). Effect of alumina on the structure and properties of Li2O–B2O3–P2O5 glasses. J. Non-Crystalline Solids.

[43] Villa M., Scagliotti M., Chiodelli G. (1987). Short range order in the network of the borophosphate glasses: A 31P NMR-MAS (Magic Angle Spinning) study. J. Non-Crystalline Solids.

[44] Feng T., Linzhang P. (1989). NMR studies of lithium borophosphate glasses. J. Non-Crystalline Solids.

[45] Jin T., Bernard G.M., Miskolzie M., Terskikh V.V., Michaelis V.K. (2018). A 11B and 31P MAS NMR study of the impact of Ca2+ and Sr2+ network modifying cations on the structure of borate and borophosphate glasses. Phys. Chem. Glass..

[46] Holland D., Feller S.A., Kemp T.F., Smith M.E., Howes A.P., Winslow D., Kodama M. (2007). Boron-10 NMR: What extra information can it give about borate glasses?. Phys. Chem. Glass..

[47] Faaborg M., Goranson K., Barnes N., Troendle E., Rice R., Chace M., Montgomry L., Koehler A., Lindeberg Z., Holland D. (2015). A 10B NMR study oftrigonaland tetrahedral borons in ring structured borate glasses and crystals. Phys. Chem. Glass..

[48] Massiot D., Fayon F., Capron M., King I., Le Calvé S., Alonso B., Durand J.-O., Bujoli B., Gan Z., Hoatson G. (2002). Modelling One- and Two-Dimensional Solid-State NMR Spectra. Magn. Reson. Chem..

[49] Medek A., Harwood J.S., Frydman L. (1995). Multiple-Quantum Magic-Angle Spinning NMR: A new method for the study of quadrupolar nuclei in solids. J. Am. Chem. Soc..

[50] Gan Z. (2000). Isotopic NMR spectra of half-integer quadrupolar nuclei using satellite transitions and magic-angle spinning. J. Am. Chem. Soc..

[51] Brow R.K., Kirkpatrick R.J., Turner G.L. (1993). Nature of alumina in phosphate glass: II. Structure of sodium aluminophosphate glass. J. Am. Ceram. Soc..

[52] Ray N.H. (1975). Study of coordination of boron in potassium borophosphate glasses by Raman spectroscopy. Phys. Chem. Glasses.

[53] Iuga A., Ader C., Gröger C., Brunner E. (2006). Applications of Solid-State 31P NMR Spectroscopy. Annual Reports on NMR Spectroscopy.

[54] Tricot G. (2019). Mixed Network Phosphate Glasses: Seeing Beyond the 1D 31P MAS-NMR Spectra With 2D X/31P NMR Correlation Maps. Annual Reports on NMR Spectroscopy.

[55] Gullion, T, Schaefer, J (1989). Rotational echo double-resonance NMR. J. Magn. Reson..

[56] Bertmer M., Eckert H. (1999). Dephasing of spin echoes by multiple heteronuclear dipolar interactions in rotational echo double resonance NMR experiments. Solid State Nucl. Magn. Reson..

[57] Lesage A., Sakkallariou D., Steurnagel S., Emsley L. (1998). Carbon-proton chemical shift correlation in solid-state NMR by through-bond multiple quantum spectroscopy. J. Am. Chem. Soc..

[58] Caravatti P., Bodenhausen G., Ernst R. (1982). Heteronuclear solid-state correlation spectroscopy. Chem. Phys. Lett..

[59] Hartmann S.R., Hahn E.L. (1962). Nuclear Double Resonance in the Rotating Frame. Phys. Rev..

[60] Gan Z. (2007). 13C/14N heteronuclear multiple-quantum correlation with rotary resonance and REDOR dipolar recoupling. J. Magn. Reson..

[61] Tricot G., Lafon O., Trébosc J., Delevoye L., Méar F., Montagne L., Amoureux J.-P. (2011). Structural characterisation of phosphate materials: new insights into the spatial proximities between phosphorus and quadrupolar nuclei using the D-HMQC MAS NMR technique. Phys. Chem. Chem. Phys..

[62] Tricot G., Trébosc J., Pourpoint F., Gauvin R., Delevoye L. (2014). ChemInform Abstract: The D-HMQC MAS-NMR Technique: An Efficient Tool for the Editing of Through-Space Correlation Spectra Between Quadrupolar and Spin-1/2 (31P, 29Si, 1H, 13C) Nuclei. Chemin-.

[63] Edén M., Zhou D., Yu J. (2006). Improved double-quantum NMR correlation spectroscopy of dipolar-coupled quadrupolar spins. Chem. Phys. Lett..

[64] Yu Y., Stevensson B., Edén M. (2018). Direct Experimental Evidence for Abundant BO4–BO4 Motifs in Borosilicate Glasses From Double-Quantum 11B NMR Spectroscopy. J. Phys. Chem. Lett..

[65] Wang S., Stebbins J.F. (1998). On the structure of borosilicate glasses: a triple-quantum magic-angle spinning 17O nuclear magnetic resonance study. J. Non-Crystalline Solids.

[66] Stebbins J.F., Oglesby J.V., Xu Z. (1997). Disorder amog network-modifier cations in silicate glasses: New constraints from triple quantum 17O NMR. Amer. Min..

[67] Aguiar P.M., Michaelis V.K., McKinley C.M., Kroeker S. (2013). Network connectivity in cesium borosilicate glasses: 17O multiple-quantum MAS and double-resonance NMR. J. Non-Crystalline Solids.

[68] Nicoleau E., Angeli F., Schuller S., Charpentier T., Jollivet P., Moskura M. (2016). Rare-earth silicate crystallization in borosilicate glasses: Effect on structural and chemical durability properties. J. Non-Crystalline Solids.

[69] Lacomb M., Rice D., Stebbins J.F. (2016). Network oxygen sites in calcium alumioborosilicate glasses: Results from 17O{27Al} and 17O{11B} double resonance NMR. J. Non-Cryst. Solids.

[70] Ohkubo T., Tsuchida E., Deguchi K., Ohki S., Shimizu T., Otomo T., Iwadate Y. (2018). Insights from ab initio molecular dynamics simulations dor a multicomponent oxide glass. J. Amer. Ceram. Soc..

[71] Uesbeck T., Eckert H., Youngman R., Aitken B. (2016). The structure of borophosphosilicate pure network former glasses studied by multinuclear NMR spectroscopy. J. Phys. Chem. C.

